# Taxonomic revision and distribution of herbaceous *Paramollugo* (Molluginaceae) in the Eastern Hemisphere

**DOI:** 10.3897/phytokeys.73.10365

**Published:** 2016-10-21

**Authors:** Alexander P. Sukhorukov, Maria Kushunina

**Affiliations:** 1 Department of Higher Plants, Biological Faculty, Lomonosov Moscow State University, 119234, Moscow, Russia; 2 Department of Plant Physiology, Biological Faculty, Lomonosov Moscow State University, 119234, Moscow, Russia

**Keywords:** Madagascar, Mollugo, New Caledonia, new species, seed characters, taxonomy

## Abstract

The genus *Paramollugo* with the type species *Paramollugo nudicaulis* (≡*Mollugo nudicaulis*) has recently been described after molecular investigations. Here we report two new endemic Malagasy species: *Paramollugo simulans* and *Paramollugo elliotii*, and transfer a forgotten New Caledonian endemic *Mollugo digyna* to *Paramollugo* (*Paramollugo digyna*). Consequently, the number of *Paramollugo* species in the Eastern Hemisphere is increased from three to six. Almost all genus representatives (except *Paramollugo nudicaulis*, which has a wide distribution in Southern Asia, Arabia and tropical Africa) are endemic to Madagascar, Somalia, or New Caledonia. Since the type of seed coat ornamentation is crucial for species delimitation, a diagnostic key with new taxonomically significant carpological characters and other new traits is provided for all the herbaceous *Paramollugo*. The distribution patterns of *Paramollugo nudicaulis* s.str., *Paramollugo simulans* and *Paramollugo elliotii* are presented.

## Introduction

The new genus *Paramollugo* Thulin has recently been split from *Mollugo* s.l. after molecular investigations (Thulin et al. 2016). It comprises five herbaceous species and one small shrub, the Malagasy *Paramollugo decandra* (ex-*Mollugo decandra*). Herbaceous *Paramollugo* species clearly differ from all other Molluginaceae by having rosulate leaves only. The inflorescences in herbaceous *Paramollugo* are bracteose, dichasial or trichasial (‘pseudo-dichotomous’), with long inflorescence stems (Batenburg et al. (1984) sub *Mollugo*) and numerous sterile branches up to 2.5 cm long, which are rarely seen in Molluginaceae. The seeds of some species have only recently been investigated (Hassan et al. 2005 sub *Mollugo*; Sukhorukov and Kushunina in press), and their morphology and ornamentation support the data obtained from a molecular phylogeny study (Christin et al. 2011), which suggests that *Paramollugo angustifolia* and *Paramollugo navassensis* are close to *Paramollugo nudicaulis*. The following herbaceous species were accepted after establishing the new generic name (Thulin et al. 2016): *Paramollugo nudicaulis* (type species) with wide distribution in the Eastern Hemisphere, *Paramollugo angustifolia* known only from the type locality in south-central Somalia (Gilbert and Thulin (1993) sub *Mollugo angustifolia*), *Paramollugo cuneifolia* (Grisebach (1866) sub Mollugo nudicaulis var. cuneifolia), *Paramollugo deltoidea* from Cuba (Léon (1950) sub *Mollugo deltoidea*), and *Paramollugo navassensis* from several Caribbean islands (Ekman (1929) sub Mollugo nudicaulis var. navassensis). Morphologically, these species differ in life form (annual or perennial), leaf shape and sometimes leaf width (Gilbert and Thulin 1993; Thulin et al. 2016).

We have previously shown that seed morphology and ultrasculpture are very useful for the diagnostics and taxonomy of major groups within the former *Mollugo* s.l., and that the ‘*Mollugo nudicaulis*’ group (=*Paramollugo*) needs further taxonomic studies (Sukhorukov et al. 2016; Sukhorukov and Kushunina in press). Here we provide a closer look at the taxonomy of the herbaceous *Paramollugo* taxa and discuss new morphological characters essential for delimiting the taxa within this complicated group.

## Materials and methods

The revision of herbarium material was undertaken in the collections B, BM, BR, E, HUJ, K, LE, M, MHA, MSB, MW, P, PRA, W, and WU (acronyms follow Thiers 2016+). In the herbaria visited in 2015–early 2016, prior to the publication of the new taxonomic revision of Molluginaceae (Thulin et al. 2016), the specimens belonging to the new *Paramollugo* species described here were labelled by us as *Mollugo* (including the holotype specimens). The distribution maps of *Paramollugo nudicaulis*, *Paramollugo angustifolia*, *Paramollugo elliotii* and *Paramollugo simulans* are based upon the specimens examined in the herbaria listed above. These specimens are cited in the Results and Discussion section for the new taxa and combination or in the Appendix 1 for *Paramollugo nudicaulis* and *Paramollugo angustifolia*. Countries are listed in alphabetical order.

The seed surface of *Paramollugo* was examined using scanning electron microscopy (SEM; JSM–6380, JEOL Ltd., Japan) at 15 kV after sputtercoating with gold-palladium. Fallen fruits (1–2) from the following vouchers were used for SEM:

*Paramollugo digyna* (Montrouz.) Sukhor.: NEW CALEDONIA. Canala, [without date] anonym 236 (BM); 1861–1867, *Viellard 120* (K); 1870, *Pancher 236* (K, P04583449); Lower slopes of Mt. Kafeate, 30 March 1956, *H.S. McKee 4228* (E, K);

*Paramollugo elliotii* Sukhor.: MADAGASCAR. Ambongo, 14 February 1841, *M. Pervillé 643* (P04582808); Mailake prov., February 1890, *M. Douillot s.n.* (P04582888); Baie de Baly, January 1905, *H. Perrier de la Bathie 9211* (P04582813); Majunga, 27 April 1912, *Afzelius s.n.* (K); Environs de Majunga, December 1924, *H. Humbert & H. Perrier de la Bathie 4020* (P04582873);

*Paramollugo nudicaulis* (Lam.) Thulin: BHUTAN. Tashigang distr., Dangme Chu valley, 3000 ft, 17 August 1915, *Cooper & Bulley 4498* (BM); CENTRAL AFRICAN REPUBLIC. Bossangoa, 12 November 1981, *Fay s.n.* (K); MADAGASCAR. [without precise location and date] *R. Capuron s.n.* (P04582819); Nasi-Be, May 1879, *Hildebrandt 2974* (LE); Tulear, 6 January 1988, *Phillipson 2800* (K); SENEGAL. Basse-Casamance, October 1988, *van den Berghen 8188* (BR0000017458177); UGANDA. Singo co., 13 April 1970, *Katende 103* (K); TOGO. Lomé to Cacaveli, 14 April 1978, *Hakki et al. s.n*. (B); YEMEN. Socotra, 4 km SE of Hadiboh, 19 February 1989, *Miller et al. 8225* (E);

*Paramollugo simulans* Sukhor.: MADAGASCAR. Vaingaindrano, [without date] *Scott Elliot 2257* (BM, K, E); Majunga, October 1924, *H. Humbert 4079* (P04582884); Fenerive [Fenoarivo], 7 September 1959, *Rauh 169* (M); Majunga province, 25 February 1985, *Dorr et al. 3810* (K); Beloka, 1 December 1989, *Dupuy 2448* (K); near Andranokoditra, 7 February 1990, *Bogner 2084* (M); Toliara, Belalanda, 17 March 2006, *R. Ranaivojaoana et al. 1442* (K).

## Results

**Figure 1.**
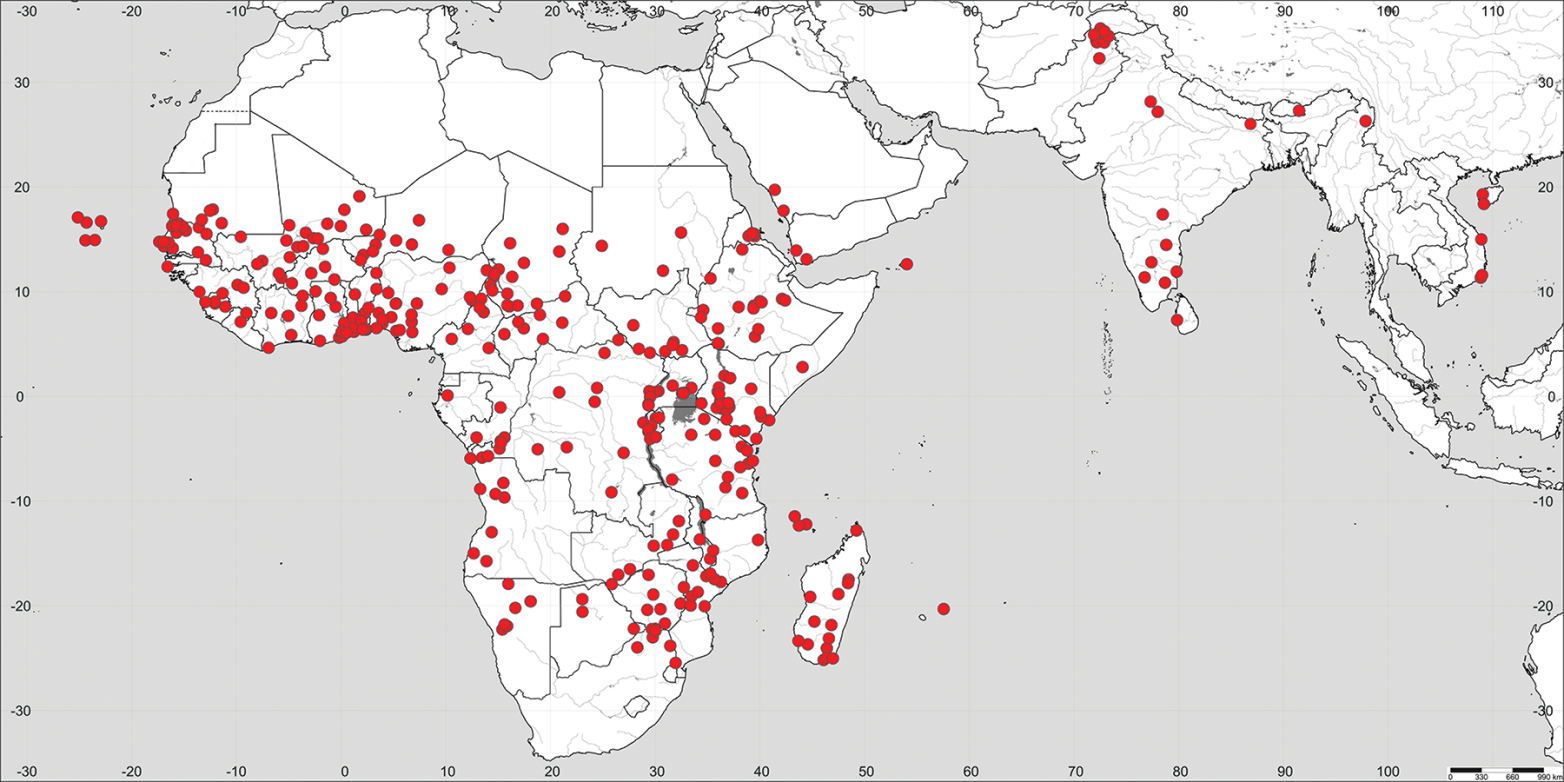
Distribution of *Paramollugo nudicaulis* in the Eastern Hemisphere.

Six *Paramollugo* species in the Eastern Hemisphere (*Paramollugo angustifolia*, *Paramollugo decandra*, *Paramollugo digyna*, *Paramollugo elliotii*, *Paramollugo nudicaulis*, *Paramollugo simulans*) are accepted in the present paper, instead of the three previously recognized species (*Paramollugo angustifolia*, *Paramollugo decandra*, and *Paramollugo nudicaulis*). The core species, *Paramollugo nudicaulis* s.str., is found to be widely distributed in the tropics of the Eastern Hemisphere as a weed species (see Fig. 1 and Appendix 1 for vouchers), and this conclusion is supported by morphological and carpological (including SEM) comparison of the observed specimens. *Paramollugo* is best represented in Madagascar, where four species are found: herbaceous *Paramollugo simulans*, *Paramollugo elliotii*, *Paramollugo nudicaulis*, and shrubby *Paramollugo decandra*. Except for *Paramollugo nudicaulis*, all species are endemics of this island. Another enigmatic taxon described as *Mollugo caespitosa* (Scott Elliot 1891) and included in the list of Madagascan endemics (Clifton 2012) is now synonymized with *Mollugo decandra* (Sukhorukov et al. 2016).

**Figure 2.**
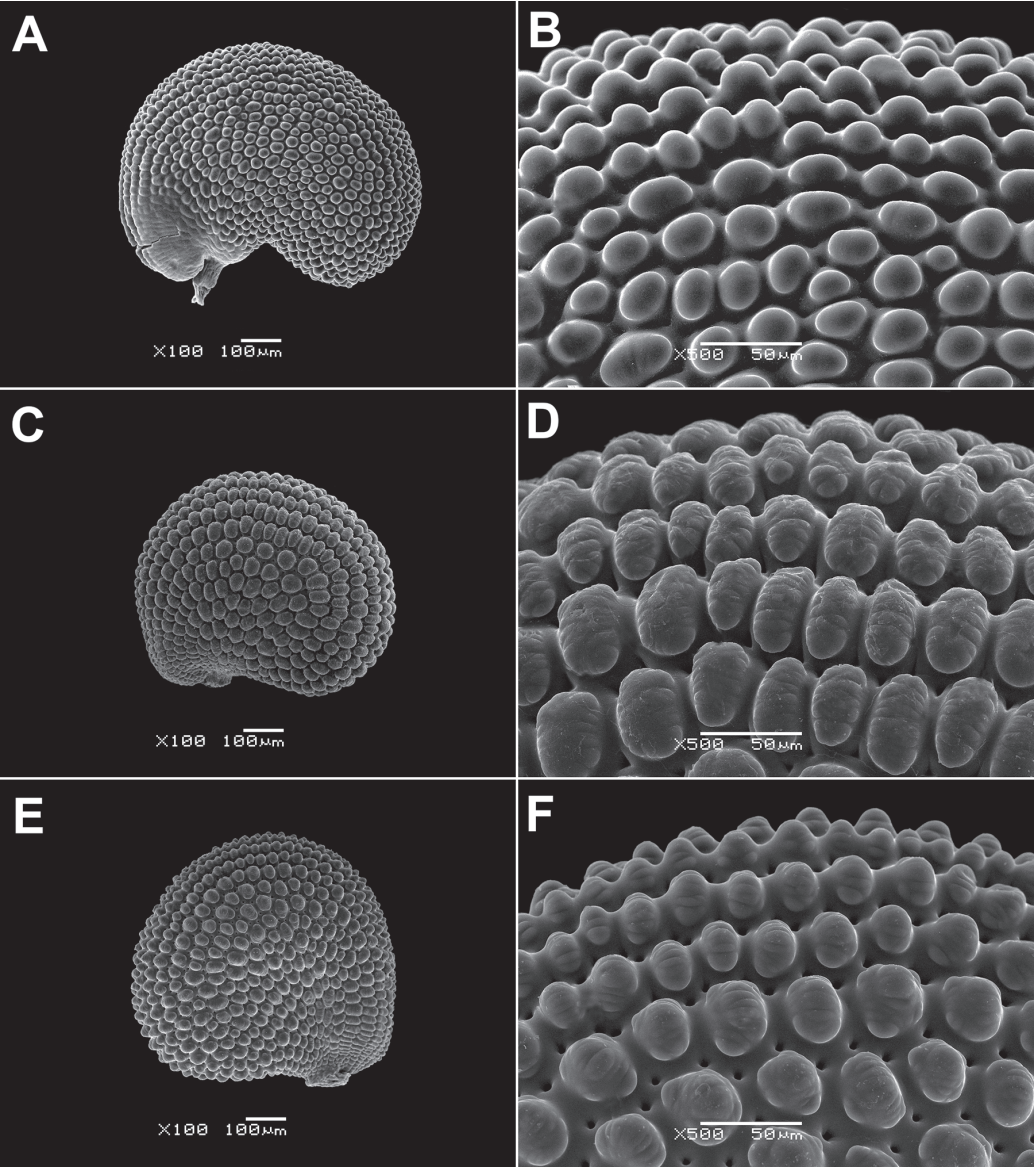
SEM micrographs of the seed surface. **A, B** *Paramollugo decandra* **C, D** *Paramollugo digyna* **E, F** *Paramollugo nudicaulis*.

**Figure 3.**
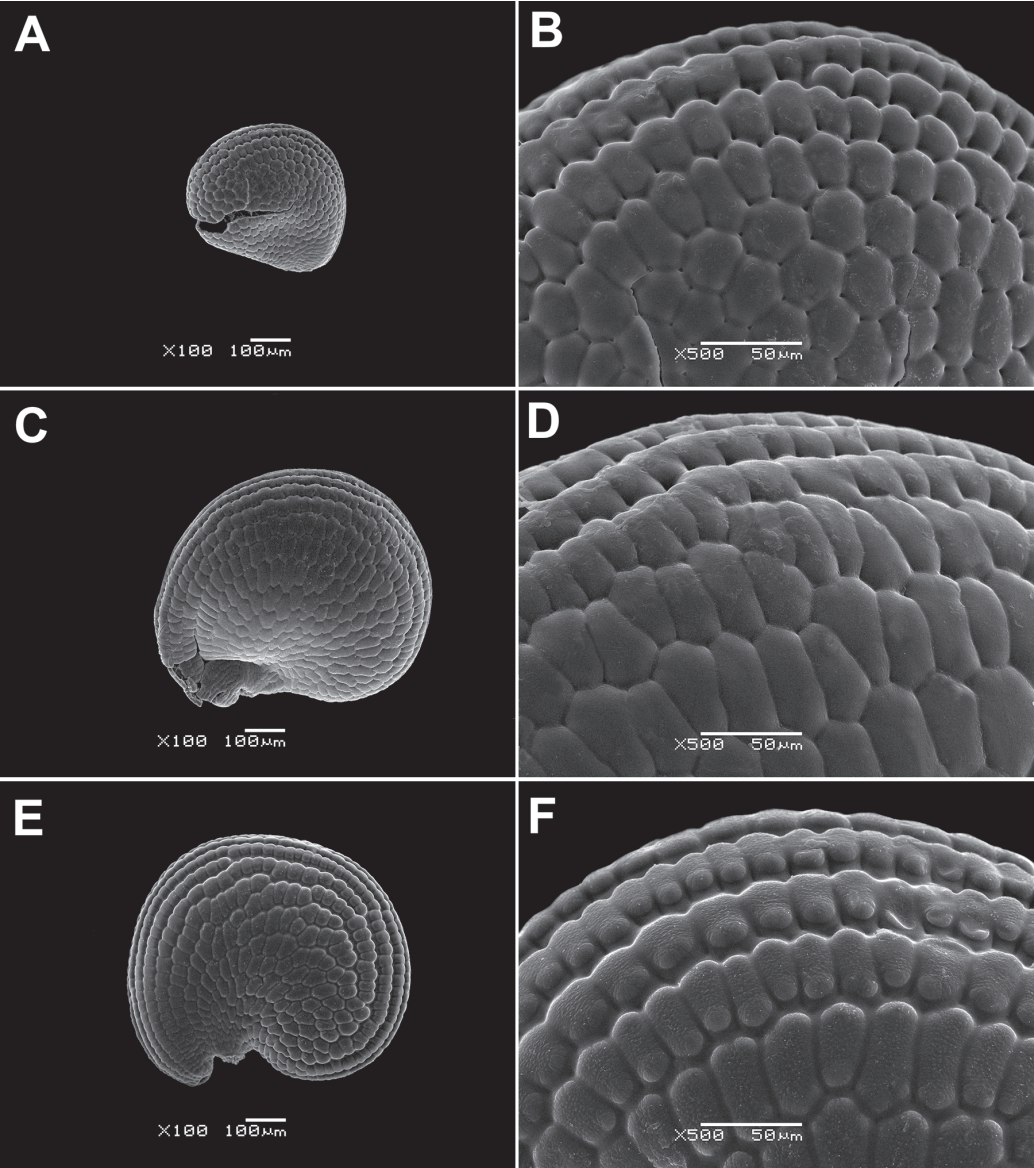
SEM micrographs of the seed surface. **A, B** *Paramollugo angustifolia* **C, D** *Paramollugo elliotii* **E, F** *Paramollugo simulans*.

The genus *Paramollugo* in the Eastern Hemisphere is one of the most morphologically difficult groups in Molluginaceae. We found that the following reproductive characters are useful for delimiting the *Paramollugo* species: size of the perianth segments, length of the anthers, number of stylodia and locules in the capsule, and seed shape and ornamentation. Seed surface is either papillate (*Paramollugo digyna*, *Paramollugo decandra*, *Paramollugo nudicaulis*; Fig. 2) or colliculate (*Paramollugo angustifolia*, *Paramollugo elliotii*, *Paramollugo simulans*; Fig. 3), while the secondary ornamentation of the testa cells is cross-striate in *Paramollugo nudicaulis* and warty in *Paramollugo digyna*, or absent in *Paramollugo angustifolia*, *Paramollugo decandra*, *Paramollugo elliotii*, and *Paramollugo simulans*. All six species are united in having small pits in the boundaries between seed coat cells (but the pits are hardly discernible in *Paramollugo elliotii*).

### Key to the delimitation of herbaceous *Paramollugo* species in the Eastern Hemisphere

*Paramollugo decandra* is not included in the text; it is a small shrub (this character differentiates this species from all other *Paramollugo* in this region, which are herbs)

**Table:** 

1	Seeds with well-visible papillate ornamentation and warty or striate outgrowths of the testa cells	**2**
–	Seeds without distinct papillae	**3**
2	Annual; leaves obovate or oblong; capsules three-valved; outer wall of the testa cells striate, without warty outgrowths	***Paramollugo nudicaulis*** (tropical Africa and Asia)
–	Annual or perennial herb; leaves reniform or almost triangular; capsules usually two-valved; outer wall of the testa cells with several or numerous warty outgrowths	***Paramollugo digyna*** (New Caledonia)
3(1)	Perennial herb with numerous (25–50) linear or lanceolate sessile leaves to 5 mm wide, not divided into petiole and lamina; anthers 0.55–0.70 mm; outer perianth segments 2.6–3.0 mm	***Paramollugo elliotii*** (Madagascar)
–	Annuals with 5–20 leaves of different shape; anthers 0.25–0.35 mm; perianth segments at fruiting stage not exceeding 2.5 mm in length	**4**
4	Leaves linear, to 2 mm wide; seeds 0.35–0.45 mm in diameter	***Paramollugo angustifolia*** (Somalia)
–	Leaves usually oblong to obovate, to 12 mm; seeds 0.55–0.7 mm in diameter	***Paramollugo simulans*** (Madagascar)

#### Paramollugo simulans

Taxon classificationPlantaeCaryophyllalesMolluginaceae

Sukhor sp. nov.

urn:lsid:ipni.org:names:77158272-1

##### Holotype.

**Figure 4.**
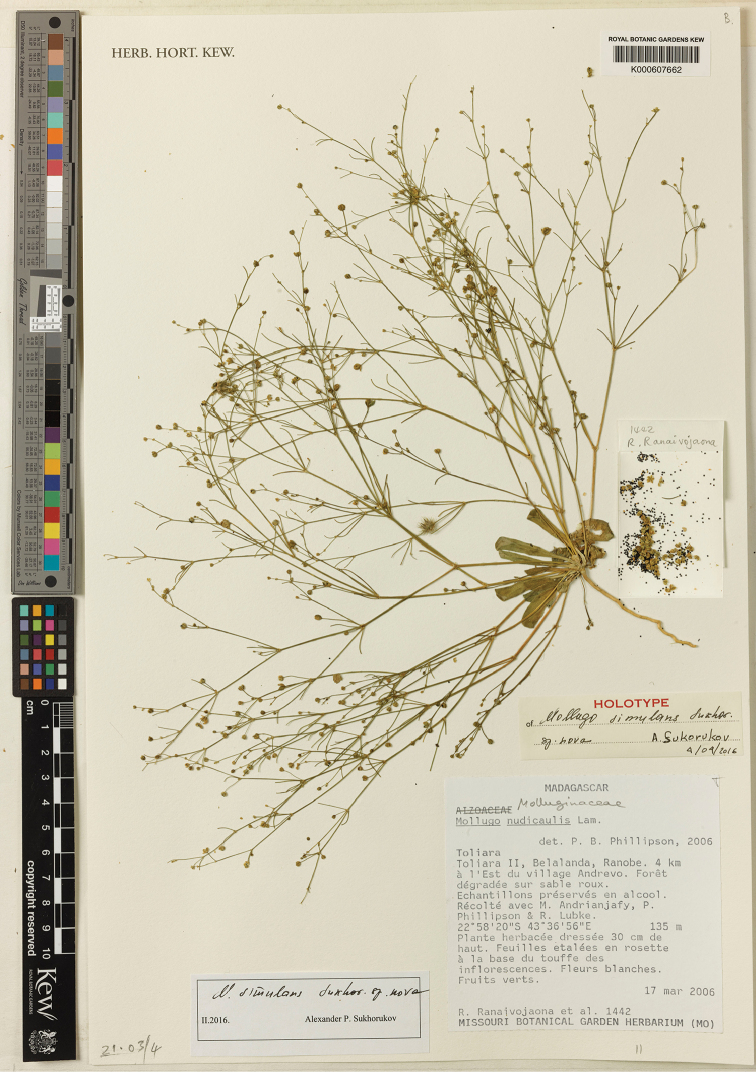
Holotype of *Paramollugo simulans* sp. nov. (K!).

MADAGASCAR, [Atsimo-Andrefana region], Toliara [district], Belalanda, Ranobe, 4 km a l’Est du village Andrevo, 22°58'20"S, 43°36'56"E, 135 m, 17 March 2006, *R. Ranaivojaoana et al. 1442* (holotype K000607662; isotype BR0000000514054, P05196698). (Fig. 4).

##### Diagnosis.

*Paramollugo simulans* is morphologically similar to *Paramollugo nudicaulis* but differs by having colliculate seed surface (testa cells slightly convex, not papillae-like) with no striate secondary cell ornamentation. Some forms of *Paramollugo simulans* with linear leaves look like *Paramollugo angustifolia*, but they reliably differ by the seed size (0.55–0.7 mm and 0.35–0.45 mm, respectively).

##### Description.

Annual to 25(30) cm; leaves (5–9)10–25, rosulate, rather thin, obovate, narrowly oblong, sometimes lanceolate, 20–50 × 2–12 mm, continuously tapered in petiole (up to 20 mm) or rarely not clearly divided into petiole and blade if lanceolate or linear, glabrous or shortly papillate; stems many (rarely 1 in underdeveloped exemplars), branched in the middle part with many lateral branches forming loose inflorescence with semi-spherical habit, always with short (up to 20 mm) sterile branches (not carrying flowers); peduncles to 15 mm, basally with white or brownish bract to 1.5 mm; flowers 3–4 mm in diameter when open; perianth segments 1.3–1.7 mm long in flowering, 2–2.4 mm long in fruiting, ovoid, green from abaxial side, white or pinkish inside; stamens 5 to 10, anthers elliptic, 0.30–0.35 mm; capsule 3-valved, ovoid, 1.5–2.3 mm, contains (8)10–18 seeds; seeds are reniform, dark red or almost black, 0.55–0.7 × 0.6 × 0.3 mm, with colliculate ultrasculpture.

##### Ecology.

Sandy, rocky, ruderal sites or dry forests at elevations 0–1700 m.

##### Flowering and fruiting.

November–June.

##### Additional specimens examined.

MADAGASCAR: **Alaotra-Mangoro region**: Moramanga, Andasibe, Berano, Ambatovy forest, Analamay, 18°49'05"S, 48°19'25"E, 1145 m, 24 February 2005, *P. Antilahimena et al. 3464* (P05196697); **Androy region**: Beloha, 25°05'S, 45°10'E, alt. 125 m, deciduous thicket with Indigofera & Crotalaria, locally common, 1 December 1989, *Dupuy 2448* (K); **Anosy region**: Fort Dauphin [Tolanaro], St. Luce, 1 May 1989, *N. Dumetz 720* (P04582891); **Atsinanana region**: Vaingaindrano, [without date] *Scott Elliot 2257* (BM, K, E); Vatomandry distr., foret de copaliers, February 1904, *J. Guillot 69* (P04582879); bei Andranokoditra, entlang der Lagune wachsend, 7 February 1990, *Bogner 2084* (M); Toamasina, Fokontany Ampitambe, Ambatovy, 18°50'04"S, 48°17'46"E, 1107 m, 15 February 2005, *H. Razanatsoa et al. 121* (P05196696); **Boeny region**: Environs de Majunga, 1908, *H. Perrier de la Bathie 5233* (P04582816); Majunga [Mahajanga], 27 April 1912, *K. Afzelius s.n.* (K); Environs de Majunga, October 1924, *H. Humbert 4079* (P04582884); Majunga province, poste forestier d’Ampijoroa (Jardin Botanique), foret seche sur sable, 16°14'S, 46°28'E, 25 February 1985, *L.J. Dorr, L.C. Bernett & M.R. Cheek 3810* (BR0000017461078, K); Mahajanga, Analalava, appr. 25 km E of Analalava towards Antsohihy, 14°46'03"S, 47°47'53"E, 178 m, low dry forest, 21 June 2008, *M. Andriamahay & S. Rakotoarisoa 0466479* (K); **Haute Matsiatra region**: Fenerive [Fenoarivo], 7 September 1959, *Rauh 169* (M); Fianarantsoa, Fivondroana, Nosy Varika, Firaisana, Ambahy, 20°47'49"S, 48°28'58"E, 10 m, 23 April 2004, *R. Razakamalala, R. Ranaivojaona & Z. Rogers 1208* (P05196706); **Ihorombe region**: Menarahaka, Ihosy, 700–800 m, vestiges de foret tropophile sur terrains siliceux, 1955, *H. Humbert 28574* (P04582868); plateau et vallees de l’Isalo a l’Ouest de Ranohira, gres et sables siliceux, 1955, *H. Humbert 28729* (P04582852; P04582855); **Melaky region**: Beanka, East Ambinda, 18°02'24"S, 44°28'31"E, 222 m, 2 April 2013, *L. Ranaivoarisoa 054* (BR0000015215840); **Sofia region**: Marokitraro, 27 December 1922, *Decary 1392* (P04582900); **Vakinankaratra region**: Antananarivo, Antsirabe, 1669 m, exposed rock, 26 February 2008, *M. Andriamahay & S. Rakotoarisoa 0452506* (K); **Vatovavy-Fitovinany region**: Mananjary prov., 1909, *F. Geay 7636 & 7790* (P04582870; P04582871; P04582872); Ambila, 7 May 1928, *M. Decary 6422* (K).

##### General distribution.

**Figure 5.**
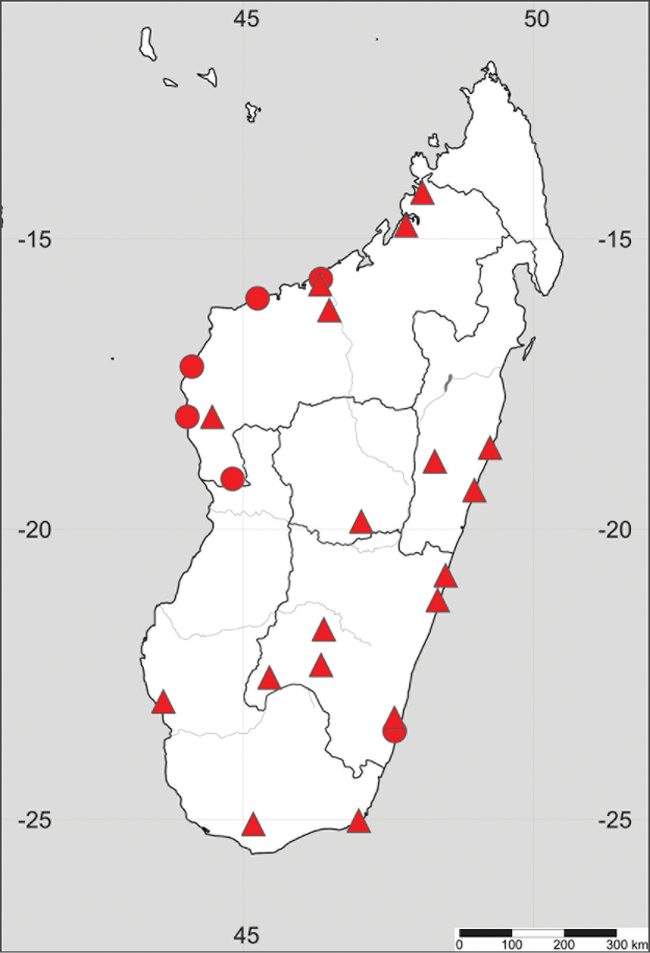
Distribution of *Paramollugo simulans* (triangles) and *Paramollugo elliotii* (dots).

Endemic to Madagascar (Fig. 5).

##### Conservation status.

Although a rather limited number of specimens is available, it is clear that *Paramollugo simulans* can occupy disturbed habitats and is therefore unlikely to be at risk of extinction. Following the IUCN (2016) guidelines, this species should be categorized as not threatened.

##### Etymology.

The specific epithet “simulans” means “imitating” and refers to the morphological similarities of the new species and *Mollugo nudicaulis*.

#### Paramollugo elliotii

Taxon classificationPlantaeCaryophyllalesMolluginaceae

Sukhor sp. nov.

urn:lsid:ipni.org:names:77158273-1

##### Diagnosis.

*Paramollugo elliotii* is morphologically similar to both *Paramollugo nudicaulis* and *Paramollugo simulans*, but differs by perennial life form, narrow (linear to oblanceolate), (sub)sessile leaves, larger anthers (0.55–0.70 mm), and larger outer perianth segments (2.6–3 mm) at the fruiting stage (*Paramollugo nudicaulis* and *Paramollugo simulans* are annuals, with broader, usually petiolate leaves, anthers of 0.25–0.30 mm and smaller perianth to 2.4 mm long).

##### Holotype.

**Figure 6.**
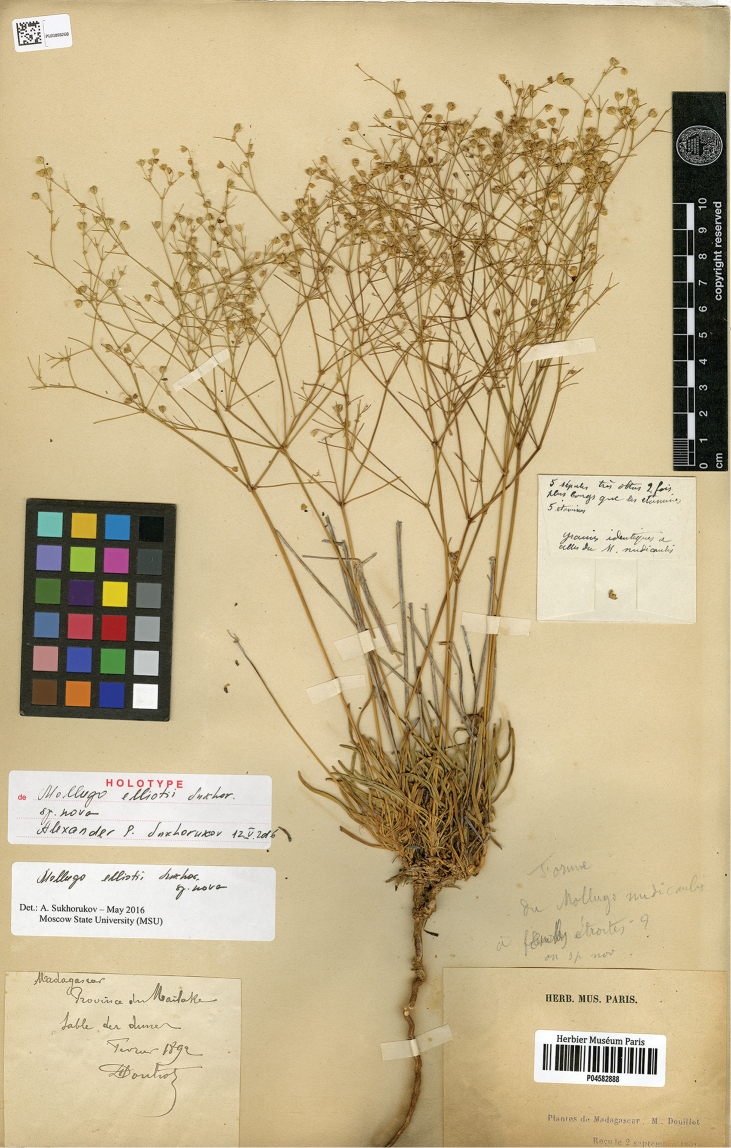
Holotype of *Paramollugo elliotii* sp. nov. (P04582888!).

MADAGASCAR, province du Mailake [Melaky region], sable des dunes [sand dunes], Fevrier [February] 1890, *M. Douillot s.n.* (P04582888!). (Fig. 6).

##### Description.

Perennial herb to 35–40 cm; leaves 25–50, rosulate, thick, linear to oblanceolate, (10)20–60 × 0.5–5.0 mm, sessile or not clearly divided into petiole and lamina, glabrous; stems several, stout, branched in the middle part and forming loose semi-spherical inflorescence with numerous lateral branches, sterile (up to 30 mm) branches always present; peduncles to 15 mm long, basally with white or brownish bract 0.7–1.2 mm long; opened flowers 3.5–4.5 mm in diameter; perianth segments ovoid, green on the abaxial side, white or pink inside, 1.5–2.0 mm in flowering, unequally enlarging at fruiting stage (three outer segments 2.6–3.0 mm long, two inner 2.3–2.6 mm long); stamens 5, anthers elliptic, 0.55–0.7 mm long; capsule 3-valved, ovoid, ca. 2 mm long, with (9–14)15–21 seeds; seeds reniform, black, 0.55–0.7 × 0.5 × 0.3 mm, with colliculate surface.

##### Ecology.

All examined specimens were collected on the sand dunes near the sea at lower elevations 0–300 m. We assume that *Paramollugo elliotii* is a psammophilous species.

##### Flowering and fruiting.

All examined specimens (collected in January, February, July, September or December) bear both flowers and fruits. However, the exact phenology has to be investigated more precisely, since it can depend on the geographical latitude and other conditions.

##### Additional specimens examined.

MADAGASCAR: **Atsimo**-**Atsinanana region**: Ambongo, 14 February 1841, *M. Pervillé 643* (K, P04582808; P04582809; P04582810); **Melaky region**: province de Mailake, Namela, dunes, February 1892, *M. Douillot s.n.* (P04582869, P04582886); Baie de Baly, January 1905, *H. Perrier de la Bathie 9211* (P04582813; P04582814); along Manambolo river, 19°09'S, 44°49'E, 50 m, 2 December 1996, *C.C.H. Jongkind 3342* (BR0000000555871); **Boeny region**: Majunga, 27 April 1912, *Afzelius s.n.* (K); Majunga, dunes, 24 December 1920, *H. Poisson 44* (P04582831); Environs de Majunga, dunes, December 1924, *H. Humbert & H. Perrier de la Bathie 4020* (K, P04582873) & *2020* (P04582874; P04582877);

##### General distribution.

Endemic to Madagascar (Fig. 5).

##### Conservation status.

All except one specimen are nearly a hundred years old or even older, and no information is available on the current extent of the wild populations of this species. Therefore *Paramollugo elliotii* is given a Data Deficient (DD) status (IUCN 2016).

##### Etymology.

The specific epithet is given after after George Francis Scott Elliot (1862–1934), a British botanist who provided significant contributions to the flora of Madagascar.

#### Paramollugo digyna

Taxon classificationPlantaeCaryophyllalesMolluginaceae

(Montrouz.) Sukhor comb. nov.

urn:lsid:ipni.org:names:77158274-1

Mollugo digyna Basionym; Montrouz., Ann. Acad. Roy. Sci. Lyon, Sect. Sci. 10: 179 (1860).

##### Neotype

(Sukhorukov, selected here). NEW CALEDONIA. West face of Massif de Koniambo, 300 m, iron-serpentine scrub on track through “maquis”, 11 October 1963, *P.S. Green 1287* (K001045648!).

##### Discussion.

**Figure 7.**
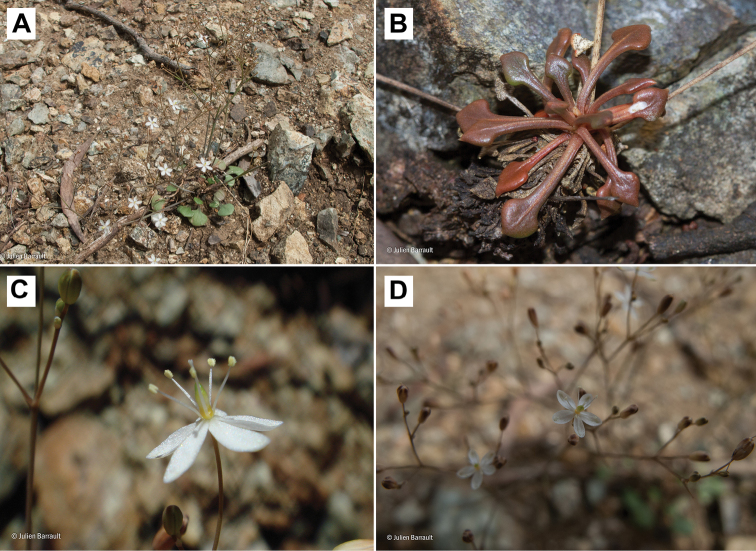
*Paramollugo digyna*. **A** general habit **B** leaf rosette **C** flower **D** inflorescence. Photographs by Julien Barrault.

This taxon described by X. Montrouzier was completely forgotten and cited as *Mollugo nudicaulis* in the treatments of the New Caledonian flora (e.g., Guillaumin 1948, Rageau 1973). However, *Paramollugo digyna* is easily recognized by unique reniform or even triangular leaf blades (Fig. 7), and by a tendency to become a short-lived perennial (in contrast to annual *Paramollugo nudicaulis*). Unfortunately, many specimens were collected without the basal portion or in their first growing season, and it is still unclear which life form is predominant in *Paramollugo digyna*. Neither perennial habit nor unusual leaf shape were mentioned in the protologue (Montrouzier 1860), but the author indicated the presence of two-valved capsules. We also found that the specimens collected in New Caledonia have two- or rarely three-valved capsules with 8 or fewer seeds, in contrast to other herbaceous *Paramollugo*, which are characterized by capsules with three locules and up to 30 seeds. The outer walls of the testa cells are elongated, almost rectangular, with secondary warty outgrowths. A similar seed surface was observed in the Caribbean *Paramollugo navassensis* and is clearly distinct from *Paramollugo nudicaulis* (Sukhorukov and Kushunina in press, sub *Mollugo*).

No original material of *Paramollugo digyna* was traced. The herbarium of X. Montrouzier, who described *Mollugo digyna*, is located in University of Montpellier herbarium (MPU), with some duplicates in the National Museum of Natural History, Paris (P). Only one specimen of *Paramollugo digyna* was found, without a precise location (MPU-310526). No specimens are cited in the protologue (Montrouzier 1860). The sheet found in MPU (no. 169) and dated 1866 might have been collected later, and hence cannot be considered as original material. However, the plant clearly originates from New Caledonia due to the writing on the sheet “Stylos binos semper, capsulamque bivalvam vidi!” (“stylodia always two, capsule two-valved”). Since the original material is not found (Art. 9.16 of ICN) here we select a neotype that bears a plant with perennial life form, reniform leaves and two-valved capsules as the most indicative characters of *Paramollugo digyna*.

*Paramollugo digyna* is known only from New Caledonia where it usually grows on rocky or stony substrates; locally common (Guillaumin 1964 sub *Mollugo nudicaulis*). *Paramollugo nudicaulis* s.str. is not present in New Caledonia.

##### Examined specimens

**(selected specimens).** NEW CALEDONIA: Kanala [Canala], 1859–1860, *Viellard* (BR); [no precise location] 1861–1867, *Viellard 120* (K); [no precise location] 1870, *Pancher 236* (BM, K, P04583449); Canala, 2000 ft, 17 February 1914, *Compton 1251* (BM); Lower slopes of Mt. Kafeate, 30 March 1956, stony ground, *H.S. McKee 4228* (E, K).

## Supplementary Material

XML Treatment for Paramollugo simulans

XML Treatment for Paramollugo elliotii

XML Treatment for Paramollugo digyna

## Appendix 1

Specimens of *Paramollugo nudicaulis* from the Eastern Hemisphere and *Paramollugo angustifolia* examined in the herbaria visited.

***Paramollugo angustifolia*:
**

SOMALIA, Bay region, c. 3 km SW of Dilinsoor, near Buur Dilinson, 2°24'N, 42°58'E, 20 May 1990, *Thulin, Hedrén & Abdi Dahir 7606* (K!, B! – isotypes).

***Paramollugo nudicaulis*:
**

ASIA:

BHUTAN: Tashigang distr., Dangme Chu valley, 3000 ft, 17 August 1915, *Cooper & Bulley 4498* (BM).

CEYLON: Bolawatta, 16 July 1932, *Simpson 9864* (BM); *Thwaites 2809*, without date (P04601254).

CHINA: **Hainan**: Ngai distr., Naam Shan Leng, 17 July 1932, *S.K. Lau 271* (P04601248, E); Ch’ang-kiang [Changjiang] distr., 23 May 1933, *Lau 1788* (BM, P04601252); [without precise location], 27 July 1933, *Liang 62270* (E).

INDIA: nr Madras [Chennai], 1856, Cleghorn s.n. (E); Madras [presidency], October 1885, *Gamble 18217* (BM); **Andhra Pradesh state**: Madras [Chennai], Cuddapah [Kadapa] distr., February 1886, *Gamble 10831* (K); Hyderabad, 5 August 1978, *van der Maesen 3868* (K); **Bihar state**: [no precise location and date] Clarke s.n. (LE); **Haryana state**: Palwal, June 1845, *Thomson s.n.* (K); **Karnataka state**: between Bangalore and Ramanagaram, 29 January 1952, *A. Gilli s.n.* (W); **Puducherry state**: Pondichery, 1835, *M. Perrottet s.n.* (P04601255); near Pondichery, December 1864, *L. Pierre 3662* (P04601257, P04601261); **Tamil Nadu state**: Nilghiri [Mts.], *Thomson s.n.* (BM, K); Tiruchi[rappalli] distr., Srirangam island, 13 August 1977, *Matthew 8345* (K); **Uttar Pradesh state**: Agra, 11 October 1952, *K.L. Mehra s.n*. (WU).

MYANMAR: Upper Burma [without precise location] 12 September 1990, *Huk s.n.* (E).

PAKISTAN: **Khyber Pakhtunkhwa province**: Akora, 1862, *Griffith 2471* (K); Mansehra, 29 September 1888, *Duthie 7480* (K); Swat state [district], nr Madian [Madyan], 14 August 1952, *Rodin 5508* (K); Swat, Najigram, 3 August 1953, *Stewart & Rahman 25421* (BM); Hazara distr., Khanpur, 14 September 1957, *Stewart s.n.* (E); Mansehra, Batrasi pass, 28 July 1994, *Schickhoff s.n.* (MSB-152175); Hazara, 18 km SE from Besham, 34°52'N, 72°55'E, 28 August 1995, *Dickoré 12087* (MSB-152173); Mardan distr., Malakand passs, 34°33'N, 71°55'E, 20 September 1995, *Dickoré 13353* (MSB-152174); **Punjab province**: Shahpur, 15 September 1902, *Drummond 14578* (E, K); Peshawar distr., Attock-Nizampur, 12 August 1958, *Burtt 1057* (E).

SAUDI ARABIA: [Jizan region] nr Ad Darb, Abha-Gijan road, 1 November 1985, *Collenette 5444* (E); [Al Bahah region] Bani Sharfa, 27 November 1966, *M. Mosnier 2922* (P04934292).

VIETNAM: [Binh Thuan province] Phan-Rang prov., Cana, 24 December 1923, *Poilane 9318* (K); Quang Ngai province, Dong Phu, June 1934, *Petelot 5347* (P04601246, P04601249); [Ninh Thuan province] Phan Rang, 15 July 1930, *Poilane 17872* (P04601247).

YEMEN: Socotra, 4 km SE of Hadiboh, 19 February 1989, *Miller et al. 8225* (E); Sokotra, Hadiboh Plain, 24 January 1990, *Miller et al. 10005* (E); [Al Hudaydah Governorate] Hais [Hays], 1837, *M. Botta s.n.* (P04601259); between Wusal al Ali & Beni Muftah, 17 September 1978, *Wood 2476* (E); [Lahij Governorate] Wadi Labdah, nr At Tur, 17 November 1982, *King 151* (E).

AFRICA:

ANGOLA: **Benguella province**: Membassoco, February 1942, *Faulkner A131* (BM, K); **Cuanza Norte province**: Cazengo, December 1854, *Welwitsch 2398* (BM); Ambaca, June 1855, *Welwitsch 2397* (BM, K); **Huila province**: Gambos, rio d’Areia, 14 November 1970, *A. Menezes 3538* (P04583399); **Luanda province**: Luanda, December 1857, *Welwitsch 2394* (BM); Luanda district, 1903, *M.J. Gossweiler 247* (P04583543); **Malanje province**: Pungo Andongo, February 1854, *Welwitsch 2400* (BM, K); **Namibe province**: Mosamedes, Dois Irmaos, 21 February 1956, *Mendes 1364* (BM).

BENIN: without precise locality, 1904, *Forluo s.n.* (P04583546); between Paouignan and Savalou, January 1902, *M. Poisson s.n.* (P04583577); without precise locality, 1902, *M. Poisson s.n.* (P04583579); near Agouagon, 24 May 1910, *A. Chevalier 23533* (P04583431, P04583433); Cotonou, 3 April 1962, *D. Froment 1198* (BR0000017458450); [Abomey-]Calavi, 6 April 1988, *Sinsun 608* (BR0000000054513, BR0000017458443); Bourgou province, Kalale, 10°15'N, 3°23'E, 380 m, 16 October 1988, *J. Lejoly 88/194* (BR0000000545171); Davougon, 7°10'N, 1°57'E, 9 October 1996, *M. Ayichedehou 69* (BR0000000545898); Bourgou, Malanville, Sota, 11°47'N, 3°23'E, 13 November 1998, *V. Adjakidje et al. 2575* (BR0000017458436); Yaoui (Toui-kilibo), 8°28'N, 2°37'E, 23 July 1999, *Oumorou & Lejoly 570* (BR0000000546882); Cotonou, 26 July 1999, *F. Genco 177* (BR0000000559288); Ouidah (“Whydah”), *P.E. Isert s.n.* (C10004313).

BOTSWANA: [North-West District] Kwebe Hills, 4 February 1898, *Lugard 153* (K); Central distr., Sephope [Sefhophe], 11 February 2006, *Farrington et al. 0316769* (K); [North-West District] Moremi Game Reserve, 22 February 2008, *Heath 1537* (K).

BURKINA FASO: Soum, Djibo-Barabole, 4 October 2006, *Carre et al. 0353348* (K); Ouagadougou/ Sikasso, 25 September 1958, *J.G. Adam 15436-4* (P00695360); Axe Djibo-Barabole à 8 km Ouest-Nord, 14°05'55"N; 1°42'34"E, 4 October 2006, *L. Sanou et al. BUR 451* (P00544385); Zabre, 23 July 1975, *B. Toutain 1188* (P04583418); Tengongo, 8 km S of Ouagadougou, 12°17'N, 1°31'W, 23 June 1983, *J. Lejoly 83/251* (BR0000017458337); 22 km N of Banfora, 10°48'N, 4°42'W, 12 June 1983, *J. Lejoly 83/071* (BR0000017458344); Bale, 10 km E of Boromo, 11°46'N, 2°51'W, 5 August 2005, *van der Maesen et al. 8026* (BR0000017458351).

BURUNDI (selected specimens): Rutana, 1 March 1960, *Breithof 19* (BR0000017460835); Bujumbura, 25 January 1967, *Lewalle 1525* (BR0000017460866, K, M); Bubanza prov., Randa, 3 December 1977, *Reekmans 6658* (BR0000017460941, K); Bururi prov., Mugara, 27 November 1979, *Reeekmans 8356* (K); Bubanza prov., 21 March 1980, *Reekmans 8749* (MSB-129433).

CAMEROON: **Adamawa province**: near Koti (50 km SE Banyo), 24 June 1967, *R. Letouzey 8713* (P04583439); **East province**: Bordoukou, September 1947, *anonym 1264* (P04583550); **Far North region**: about 5 km W of Maroua, c. 500 m, 2 September 1964, *W.J.J.O de Wilde & B.E.E. de Wilde-Duyfjes 2971* (P04583388, BR0000017458535); 24 August 1964, *Letouzey 6389* (P04583487); Waza (Sd Maltam), 19 September 1972, *A. Gaston 3201* (P04583377); Kaele (55 km SSE Maroua), 65 km SW of Kousseri, Kousseri-Waza road, 10 September 1984, *S. Lisowski B-985* (BR0000017458528); **North region**: Garoua, 7 September 1964, *Bounougou 114* (K, P04583486); Mayo Alim (52 km S Gouna), 9 December 1964, *J. & A. Raynal 12406* (P04557321, P04583389); Poli, 28 September 1968, *H. Jacques-Felix 8385* (P04583513); **West region**: km 18 Bafoussam-Foumbot road, 1 km E of Nchi R., 5°17'24"N, 10°18'36"E, 1000 m, 15 December 1971, *A.J.M. Leeuwenberg 8903* (BR0000017461092, M; P04583397).

CAPE VERDE: San Nicolau island, 1851, *Bolle s.n.* (K-001134117); Sal, Fogo [Sao Philippe] & Santo Antao Islands, 1875, *Loewe 37* (BM); Santa Antao island, *Löwe 37* (K-001134120); Santiago, near Praia, without date and collector (P04583443); [Santiago island] Praia, 29 November 1878, *anonym 917* (P05196643).

CENTRAL AFRICAN REPUBLIC: [Nana-Mambéré prefecture] Bouar, 1914, *Midbraed 9385* (BM, BR0000017458559); Bouar, 16 May 1967, *Audru 3520* (P04583402); [Ouham prefecture] Bossangoa, 12 November 1981, *Fay 88* (K); [Bamingui-Bangoran prefecture] Dongolo mare, 29 July 1982, *Fay 2833* (K); [Ouham-Pendé prefecture] Boguila region, 1982, *Fay et al. 5442* (K); Obo, 27 December 1963, *B. Descoings 11.954* (P04583495); [Cuvette department] Okoulou, Boundji, 22 June 1965, *A. Bouquet 1.459* (P04583496); Bambari region, Oubangui, 9 March 1928, *R.P. Tisserant 2249* (P04583514); Gribingui [river], 29 November 1902, *A. Chevalier 6387* (P04583434); [Kémo prefecture], Krebedje (Fort Sibut), 7 October 1902, *A. Chevalier 5640* (P04583435).

CHAD: **Batha region**: Mateti c. Kouki, 28 August 1968, *A. Gaston 1795* (P04583383); **Chari-Baguirmi region**: Baguirmi, 10-16 August 1903, *A. Chevalier 9571* (P04583489); **Ennedi-Ouest region**: Mortcha, Koalga, 27 September 1935, *B. Zolotarevsky, M. Murat & L. Dupont 497* (P04583447); **Logone Occidental region**: Deli, 14 December 1962, *Mosnier 1517* (P04583570); Moundou, 6 December 1964, *Audru 1898* (P04557323, P04557324); Doba, 17 August 1964, *Audru 824* (P04583393); **Kanem region**: Bochoumanga, 12 August 1964, *A. Gaston 324* (P04583450); Touil, 12 August 1964, *A. Gaston 301* (P04583569); **Mayo-Kebbi region**: Koyom, 7 July 1969, *Fotius 1517* (P04583413, P04583425); **Moyen-Chari region**: Moussafoyo, 1 July 1964, *J. Audru 114* (P04583438); c. Moussafoyo, 25 October 1968, *A. Gaston 2307* (P04583384); **N’Djamena region**: Ndjamena, 25 July 1984, *S. Lisowski E-1* (BR0000017458511); **Ouaddaï region**: Abeche, 26 June 1966, *A. Gaston 766* (P04583449).

COMOROS: [no precise location] 1884, *Humblot 438* (BM, K, P00347334); Johanna [Anjouan] Island, 1875, *Hildebrandt 1685* (BM, K, M, P00500194, WU); Moely [Moheli island], September 1847, *M. Boivin s.n.* (P00500192); Grande Comore island, 26 August 2006, *A. Kaou s.n.* (P00558066)

COTE D’IVOIRE: **Agnéby-Tiassa region**: N’douci, 20 July 2006, *A. Mangara 218* (BR0000000959397); **Bounkani region**: Bouna National Park, Tehini, 21 August 1964, *R.A.A. Oldeman 287* (BR0000017458313, K, P04583391); Kakpin, 12 August 1967, *G.J.H. Amshoff 711* (BR0000017458290); **Gbêkê region**: Baoule-Nord cercle, between Diahbo and Bouake, 9 July 1909, *A. Chevalier 22077* (P04583469); Bouake, 370 m, 1 June 1962, *A.J.M. Leeuwenberg 4275* (K, P04583386); **San-Pédro region**: Bereby [Grand-Béréby], November 1964, *G.J.H. Amshoff 646* (BR0000017458320, K); **Worodougou region**: 2 June 1909, *A. Chevalier 21823* (P04583488).

DR CONGO (selected specimens): **Bandundu province**: Lieu, Kikwit, 21 November 1990, *Masens 437* (K, BR0000017459587); **Bas-Congo province**: Moanda, [no date] *Bittremieux 287* (BR0000017458955); Madimba, 13 December 1956, *Carlier 359* (K; M); Songololo, 19 December 1959, *P. Compere 1101* (BR0000017459143); **Équateur province**: Befale, 23 June 1958, *Evrard 4277* (K); **Kasai-Oriental province**: Gandajika, 11 December 1956, L. *Liben 2081* (BR0000017459815); Bakwanga [Mbuji-Mayi], August 1958, *L. Liben 3695* (BR0000017459563); Mweka, 17 June 1958, *Decleirq 41* (BR0000017459518); **Katanga province**: Bukama, December 1948, *de Witte 4841* (K); Kongolo, 9 November 1974, *Thompson s.n.* (K); **Kinshasa province**: Leopoldstad [Kinshasa], 1922, *Achten 7* (BR0000017459020); **Kongo Central province**: Matadi, 20 December 1887, *F. Hens 324* (P04583547); **North Kivu province**: plain S of Lake Edward, 1100 m, May-June 1929, *H. Humbert 8193* (P04583509, P04583423); Katanda, September 1937, *J. Lebrun 7726* (P04583549; M); Beni, November 1954, *de Witte 11486* (K); Ndemo, Albert [Virunga] National Park, October 1956, *de Witte 13617* (K); **Orientale province**: Bili, May 1931, *Lebrun 2825* (K, P04583510); Yangambi, October 1936, *J. Vouis 2691* (P04583440); Dungu, Garamba National Park, 2 April 1950, *Noirfalis 115* (K); Opala, 13 November 1976, *S. Lisowski 43281* (BR0000017459969); **South Kivu province**: Ruzizi, 1950, *Germain 5809* (K).

ERITREA: **Northern Red Sea region**: Assaorta, August 1902, *Pappi 2663* (BM); Archico, 17 January 1952, *Stower et al. 4098* (BM, K); about 45 km on the road from Massawa to Asmara, 15°35'N, 39°15'E, 300 m, 8 November 1969, *de Wilde 4656* (BR0000017458580); **Maekel region**: Gahatli, Asmera, 31 December 1987, *Ryding & Edwards 10222* (K).

ETHIOPIA: **Afar region**: Awash National Park, 12 April 1969, *Gilbert 1210* (K); Shewa, Awash station, 8°59'N, 40°10'E, 1 August 1975, *P.C.M. Jansen 2490* (BR0000000569222); **Benishangui-Gumuz region**: between Guba-Mankush & Bambudi, 28 October 2010, *I. Friis & al. 13628* (BR0000015246189); **Gambela region**: Gambela, 17 May 1976, *Ash 3419* (K); Gambela, 15 September 1988, *Pavlov 330* (MW); 17 km E of Punido, along the new road to Gog, 7°33'N, 34°23'E, 24 November 1995, *Friis et al. 7318* (K; BR0000017458573); **Harar region**: Harar, 17 August 1975, *Gilbert & Thulin 26* (K); **Oromia region**: Schoa, Wolliso, 1750 m, 1963, *Hildebrandt 536* (WU); Shoa prov., Wollenchiti [Welenchiti], 17 January 1972, *Gilbert 2278* (K); Sodere, 12 July 1975, *Gilbert 3972* (K); about 40 km past Harar on the road to Jijiga, 9°10'N, 42°25'E, 1400 m, 22 March 1977, *de Wilde 6389* (BR0000017458603; M); Bale region [zone], Sidambale, 10 May 1980, *Thulin et al. 3502* (K); Bale prov., Dello Mena, 29 May 1988, *Gilbert & Sebsebe 8527* (K); **SNNPR**: western bank of Omo River, to the E of Nakua volcanic chain, 5°05'N, 36°E, 500 m, 8 July 1972, *R. Bonnefille 103* (P04583421); Kaffa prov., 5 November 1984, *Gereau 1387* (K); Gamu-Gofa region, Lower Omo valley, 10 January 1998, *Friis et al. 8950* (K); **Tigray region**: Ussla valley nr Garrsarfa, 11 August 1854, *Hohenacker 2229* (BM, K, P05196646, P04583473).

GABON: [without precise locality], 7 August 1966, *Halle & Thomas 364* (P04557343).

GHANA: **Greater Accra region**: Accra, 14 March 1927, *Deighton 583* (K); Prampram, November 1951, *Morton 6080* (K); Accra, 23 September 1954, *J.O. Ankrah 20022* (P04583503); Kpong, 10 August 1958, *J. Lebrun 11329* (BR0000017458368); [Accra], Dome, near Achimota, June 1961, *F.R. Irvine 5030* (K; BR0000017458375); [Accra], Legon, 6 April 1963, *Dokozi 381* (MW); **Northern region**: Salaga, 9 July 1920, *Dalziel 57* (K); Tamale, Nyankpala Station, June 1984, *Abbiv 47245* (K); **Upper West region**: Kampaha, 2 April 1956, *Adams 3989* (K); Brong-Ahafo region, near Wenchi farm, 7°46'N, 2°06'W, 250 m, 9 May 1995, *C. Jongkind 2252* (P04583568, BR0000000515531); **Volta region**: Kpando, 1924, *Robertson 121* (BM); Adidome, 22 January 1971, *Lewis 7694* (K); **Western region**: Atwabo, 1931, *Fishlock 92* (K).

GUINEA: Kouroussa, July 1900, *M. Pobeguin 369* (P04583545); Nzerekore, S of Mt Yonon, 7°58'12"N, 9°03'W, 446 m, 26 April 2011, *C. Jongkind et al. 10331* (P06807575, BR0000017458252); Kankan, 1 November 1966, S. *Lisowski 61918* (BR0000017458269).

KENYA (selected specimens): Tsavo Natural Park, 11 April 1966, *Gillett 17268* (K); **Baringo county**: Baringo distr., 31 October 1964, *Leippert 5275* (K); Rift Valley, 6 km from Marigat to Kabarnet, 0°28'12"N, 35°58'48"E, 31 October 1964, *H. Leippert 5275* (WU); [Lake] Bogoria, 25 December 1978, *Lavranos 17043* (E); **Homa Bay county**: Kobodo, 6 January 2006, *Kirika et al. 2006/09* (K); **Isiolo county**: Modo Gash [Mado Gashi], 9 December 1977, *Stannard & Gilbert 864-889* (K); **Kajiado county**: Mt. Suswa, 4 August 1952, *Verdcourt 704* (K); Masai distr., 95 km S of Nairobi, 11 March 1977, *Hooper & Townsend 1323* (K); **Kiambu county**: Thika distr., 24 January 1970, *Thulin 336* (K); **Lamu county**: North Mombasa, way to Lamu & Witu, 1902, *Whyte s.n.* (BM); **Mombasa county**: Mombasa, 1847-1852, *Boivin s.n.* (P04583481); Mombasa, 18 October 1934, *Taylor 3923* (BM); **Muranga county**: Kora base, Tana river, 31 July 1976, *S.P. Kibuwa 2437* (BR0000000503254); Kora, 43 miles W of Garissa, 2000 m, 26 July 1976, *Tana River Expedition 29F2* (BR0000017458658); **Nairobi**, 14 May 1974, *Faden & Ng’weno 74/566* (K); **Nakuru county**: Lake Elmenteita, 16 June 1951, *Bogdan 3047* (K); Rift valley, near Eburru, August 1929, *H. Humbert 9074* (P04583511); **Narok county**: Narok distr., 5 June 1961, *Glover et al. 1566* (K); **Samburu county**: South Turkana, Kangetet, 16 May 1970, *Matthew 6221* (K); Ngoronet, 3 December 1978, *Hepper & Jaeger 7210* (K); **Taita-Taveta county**: Voi distr., Mutanda Rock, 8 February 1953, *Bally 8797* (K); **Tana River county**: Tana River National Reserve, 13 March 1998, *Luke et al. 246* (K); Bura, 10 March 1963, *N. Thairu 58* (BR0000017458665).

LIBERIA: Gbarnga distr., Suakoko, 20 April 1952, *M.L. Blickenstaff 55* (P04583499, BR0000017458276).

MADAGASCAR: Central Madagascar, August 1880, *Parker s.n.* (K); valle de Manambolo, rive Gauche, environs d’Isomono, Monts Kotriha et Isomonobe, 1933–1934, *H. Humbert 12836* (P04582864; P04582866); Ranomafana, 20 February 1964, *Peltier 4748* (P04934053); **Alaotra-Mangoro region**: D’Ambatondrazaka distr., Alaotra Lake, 1918, *Esperandieu 13* (P05196704); Ambatondrazaka, October 1938, *Cours 1262* (P04582920); **Analamanga region**: Antananarivo, [no date], *anonym s.n.* (BM); **Androy region**: Ambovombe, 12 July 1931, *Decary 9074* (P04582892); **Anosy region**: Behara, 3 September 1932, *Decary 10491* (P04582903); Mandrare, Anadabolava, December 1933, *Humbert 12403* (BM, P04582867); Fort Dauphin [Tolanaro], 1955, *Humbert & Capuron 29035* (P04582854); **Atsimo-Andrefana region**: Tulear prov., nr Beza Mahafaly Reserve, 6 Janary 1988, *Phillipson 2800* (K); **Atsimo-Atsinanana region**: Tulear, January 1947, *Humbert 19845* (P04582859); Ankazoabo, February 1967, *Morat 2550* (P04582851); Toliara prov., Tulear to St. Augustin, 5 January 1999, *De Block et al. 551* (P05196702); **Boeny region**: Mahajanga (Majunga), Ambalabao, 12°48'S, 49°14'E, 350 m, 13 March 1988, *Cheek et al. 1471* (P05196699); **Diana region**: Nosy Be Island, May 1879, *Hildebrandt 2974* (BM, M); **Ihorombe region**: Fianarantsoa, Iakora, 7 February 2005, *Andrianjafy 977* (P05196701); **Fianarantsoa region**: 21°50'11"S, 46°50'17"E, 15 March 2010, *Rakotoarivelo et al. 241* (P06807565); **Melaky region**: Tsingy de Bemaraha, north of Manambolo river, 19°9'S, 44°49'E, 50 m, 3 December 1996, *C.C.H. Jongkind 3361* (BR0000000555590, P05196710).

MALAWI: **Central region**: Chitala, 12 February 1959, *Robson 1552* (BM); **Southern region**: Shire Highlands, 6 April 1906, *Adamson 125* (K, P04583482); Port Herald [Nsanje], 260 m, 19 March 1960, *J.B. Phipps 2557* (BM, BR0000017458900); Zomba, Nselema village, 23 April 1965, *E.A. Banda 87* (BR0000017458894); South prov., 14 April 2008, *Chapama et al. 817* (K).

MALI (selected specimens): **Bamako**, June1929, *Waterlot 1199* (P04583471); **Gao region**: Gao, 12 September 1927, *Hagerup 334* (BR0000017458191, K); Tabankort, 28 August 1931, *A. Leclercq 42.647* (P04583461); Menaka, 27 November 1932, *A. Leclercq 813* (P04583459, P04583463); Gao, 1 July 1935, *de Wailly 4704* (P04583457); **Kayes region**: 5 km E Macina (c. Nioro), 22 October 1969, *G.Boudet 5973* (P04583373); **Kidal region**: Adrar [Adrar plateau], September 1969, *Popov 69/29* (BM); Adrar des Ifoghas, September 1985, *Sidiyene 9* (P04583566); **Koulikoro region**: Katibougou, 2 July 1967, *Kaden 257* (MW); Koulikoro, 13 June 1977, *Aberlin 73* (P04583390); **Mopti region**: Ngouma, 20 October 1971, *G. Boudet 7311* (P04583375); Nema, 2 August 1933, *anonym s.n.* (P04583452); Dallah, 19 June 1932, *J. Rogeon 378* (P04583458); Bandiagara, 11 June 1932, *J. Rogeon 343* (P04583460); Boni, 21 June 1899, *anonym s.n.* (P04583465); **Ségou region**: San, 29 June 1899, *A. Chevalier 1077* (P04583467; BR0000017458207); Farabougou, 8 July 1933, *anonym s.n.* (P04583453); Dioura, August 1954, *Davey 77* (K); **Sikasso region**: Sikasso, 29 October 1964, *Andronova 16* (MW); Sanzana, 7 July 1970, *N. Diarra 644* (P04583557); **Tombouctou region**: Karouassa, 30 June 1909, *M. Chudeau s.n.* (P04583472); Tilemsi, 15 September 1959, *Rossetti 144* (P04583559).

MAURITANIA (selected specimens): **Assaba region**: Tahmeire, Sortie Sud de Kiffa sur la route de Kankossa, 16°32'47’’ N, 11°24'9’’ E, 9 September 2008, *J.-N. Labat et al. 3957* (P00577618); **Brakna region**: between Bassi Nguidi and Mal, 10 October 1960, *M. Mosnier 7487* (P04583562); **Gorgol region**: Rinndiao, 1972, *Naegele s.n.* (K); Maghana, 22 August 1986, *Barriere 45* (P05196644); **Trarza region**: Tamzak, 19 October 1963, *J.G. Adam 19389-4* (P00694379, P00695683); **Tagant region**: Letfatar, 26 October 1910, *M. Chudeau s.n.* (P04583502); Moudjeru [Moudjeria], 24 June 1911, *M. Chudeau s.n.* (P04583501); Nbeika, 27 September 1997, *G. Heynemann 2940* (BR0000000938991).

MAURITIUS: Morne Brabant [Peninsula], 23 February 1973, *Gueho 15575* (K).

MOZAMBIQUE: **Manica province**: Manika & Sofala, Chimoio, 23 February 1948, *Garcia 324* (K); Dombe, Machonga, 23 November 1965, *A. Pereira & A. Marques 834* (BR0000017458924); **Nampula province**: Erati, Namapa, 21 March 1961, *Balsinhas & Marrime 304* (BM, K); **Sofala province**: Gorongosa prov. [district], 16 May 1906, *G. Vane 368* (P04583483); Gazaland, Madanda forest, 5 December 1906, *Swynnerton 2762* (BM); Chemba, November 1926, *M. Surcouf s.n.* (P04583448); **Tete province**: Tete, ca. 350 m, 5 February 1970, *A.R. Torre & M.F. Correia 17824* (P04583380); **Zambezia province**: Boroma, February 1890, *Manyharth 921* (WU); Cundine, 24 November 1908, *M. le Testu 884* (P04583533).

NAMIBIA: **Erongo region**: 18 January 1934, *Dinter 6850* (BM); Karibib, Farm Ameib, 20 March 1963, *Giess et al. 5870* (M); Karibib, 22 March 1965, *Toelken & Hardy 779* (K); Ameib, 22 March 1965, *Toelken & Hardy 779* (K); **Oshana region**: Okapsa, nr Ondangua, 8 February 1959, *de Winter & Giess 6900* (M); **Otjozondjupa region**: Erichsfelde, Eckenberg, 23 March 1956, *Volk 11910* (M); Otjiwarongo distr., Farm Okamuru, 5 March 1974, *Merxmüller & Giess 30083* (M); Grootfontein, Farm Kumkauas, 11 March 1974, *Merxmüller & Giess 30225* (M).

NIGER (selected specimens): **Agadez region**: Ighazer plain nr Agades, 9 September 1965, *Popov 35* (BM); **Maradi region**: Dakoro Ranch, 9 August 1985, *Pase 3110* (K); **Niamey**, July 1975, *P. Lavie 913* (P04583558, P04583561); **Tahoua region**: Ekrafane, 6 September 1986, *Peyre de Fabregues 4351* (P05196645); Ekrafane, 25 July 1979, *Klein 717* (P04583415); Tahoua, September 1961, *J. Koechlin 6639* (P04583454); **Téra department**: Kolo, 25 May 1942, *A. Marchal s.n.* (P04583405); **Tillaberi region**: Filingue, Toukounous, September 1961, *J. Koechlin 6577* (P04583451); Filingue, 19 August 1985, *H. Breyne 6117* (BR0000017458467); Baleyara-Filingue road, Tabla, 23 August 1987, *N. Leman 157* (BR0000017458474); **Zinder region**: Goure, 31 July 1964, *Peyre de Fabregues 556* (P05196647, P05196648).

NIGERIA: **Adamawa state**: Yola, 19 July 1909, *Dalziel 51* (K); **Anambra state**: Onitsha, 3 May 1944, *Onochie 7526* (K); Vogel Peak area, Dawo, 520 m, 19 November 1957, *Hepper 1408* (K, P04583508, BR0000017458542); **Bauchi state**: Katagum distr., 9 July 1907, *Dalziel 195* (K); River Benue, September 1910, *Talbot s.n.* (BM); Bichiki, 17 May 1921, *Lely 181* (K); **Borno state**: Dikwa, 13 December 1954, *McClintock 103* (K); **Edo state**: Okomu Forest reserve, 31 December 1947, *Brenan & Jones 8636* (BM, K, P04583493); Benin city, 31 December 1947, *J.P.M. Brenan 8636* (BR0000017458504); **Kogi state**: Kabba prov., Igala distr., between miles 6&7 of Idah-Nsukka motor road, 8 June 1963, *M.G. Latilo 47675* (K, P04583382); **Kwara state**: Lokoja distr., 22 April 1979, *Daramola 90187* (K); **Lagos state**: Lagos, 8 April 1909, *Dennett 497* (K); **Niger state**: [New] Bussa, 25 July 1965, *Cook 389* (K); **North-West state**: Kuje distr., 18 May 1973, *Eimunjeze et al. 66438* (K); **Ogun state**: Oru, 1931, *Lebrun 3585* (K); **Osun state**: Ilesha, Ipole, 11 June 1977, *Lowe 17554* (K); **Oyo state**: Iseyin rocks, 18 October 1969, *Jackson & Etukudo 8181069* (P04583381); Ibadan, 26 July 1961, *Swarbrick 2486* (E).

REPUBLIC OF CONGO: [Bouenza Department] Loudima, 12 May 1979, *Bonnin 401* (K); Brazzaville, July 1908, *A. Chevalier 4188* (P04583494); Brazzaville, November 1884, *M. Thollon 919* (P04583553); Ngatsou, 55 km NE of Brazzaville, 13 July 1985, *G. Cusset 1389* (P04583565).

RWANDA: [Eastern province] Kibungu, 10 April 1958, *Troupin 6962* (K, BR0000017460828); [Harare province] Bitshumbi, 1933, *G. de Witte 1030* (P04583548).

SENEGAL (selected specimens): **Dakar**: 18 September 1930, *J. Trochain s.n.* (P04583442); environ Dakar, 7 August 1948, *J.G. Adam 1678* (P00695351); Alamadies, 2 October 1955, *J.G. Adam 10958* (P00695339); **Diourbel region**: Bambey, 13 December 1960, *J. & A. Raynal 6680* (P04583430); **Kaolack region**: Kaolak, 1950-1951, *R.P. Berhaut 401* (P04583551); **Kédougou region**: Niokolo-Koba, 12 June 1958, *J.G. Adam 14.345* (P04583484); **Louga region**: Lagbar, 18 September 1956, *J.G. Adam 12344* (P00695336); Gallayel, 8 September 1965, *M. Mosnier 2232* (P04583392); **Saint-Louis region**: near Dagana, July 1828, *Leprieur s.n.* (P04583582); Ross-Betio [Ross Bethio], 25 August 1965, *Audru 2281* (P04583396); Dagana, 12 October 1969, *Hepper 3642* (K); Fete-Ole, 15 October 1976, *A. Cornet 536* (P04583398); NE of Lac de Guiers, 16°20'N, 15°45'W, 22 November 1984, *P. Bamps 7596* (BR0000017458153); **Tambacounda region**: Tambacounda, 1 October 1988, *C. van den Berghen 8188* (BR0000017458177); **Thies region**: Thies, 5 November 1939, *Adam 8944* (M); Nianing, 16 August 1954, *R.P. Berhaut 5370* (P04583491); **Ziguinchor region**: Basse-Casamance [National Park], October 1988, *C. Vanden Berghen 8188* (BR0000017458177).

SIERRA LEONE: Sierra Leone, 1798, *Afzelius* (LINN-HS 152.3); **Eastern province**: Njala, 25 September 1926, *Deighton 2119* (K); **Northern province**: nr Falaba, 31 March 1892, *Scott Elliot 5423* (K); Makeni, 31 August 1928, *Deighton 1276* (K); Makump, 4 May 1929, *Deighton 1413* (K); Rokupr, 20 February 1950, *Jordan 382* (K).

SOMALIA: **Bay region**: Bur Akaba [Buur Hakaba], *Gillett Hemming 24893* (K); Buur Heybe, 1985, *O’Brien s.n.* (K); Durdur, 1 June 1989, *Thulin & Mohamed 6759* (K).

SOUTH AFRICAN REPUBLIC: **KwaZulu-Natal province**: Zululand, 8 December 1971, *Pooley 1552* (E; M); **Limpopo province**: Waterberg distr., 3 March 1925, *Galpin 153* (K); Dongola Reserve, 15 March 1948, *Codd 3845* (K); Transvaal [Limpopo] prov., Kruger National Park, 4 February 1949, *Codd & de Winter 4983* (K); Transvaal, Soutpansberg, 31 January 1962, *Schlieben 9279* (K); Malalane, Kamatipoort [Komatipoort], 20 January 1966, *Hilliard & Burtt 3613* (E); Messina [Musina], 8 January 1974, *Pienaar 283* (K); **Mpumalanga province**: Komatipoort, December 1917, *Moss 607* (BM).

SOUTH SUDAN: Gondokoro, February 1910, *Mearns 3058* (BM); Mongalla, Bahr el Gebel, 7 July 1929, *Simpson 7279* (K, BM); S Sudan, 50 miles S of Rejaf, Gumbiri, September 1929, *Edwards s.n.* (BM); Bo river, 22 July 1937, *Myers 7313* (K); Zande Land, [Khor] Uze, 12 April 1939, *Wyld 500* (BM); Anglo-Egyptian Sudan, Torit, 4°94'N, 32°34'E, 24 June 1949, *Jackson 831* (BM).

SUDAN: [Kordofan state] Cordofan Mt., 3 October 1839, *Kotschy 119* (BM, BR0000017458566); Kordofan, 1837/1839, *Kotschy s.n.* (M-7419); Darfur province [region], 1000-1100 m, 1 January 1934, *Dandy 24* (BM); [Khartoum state] Omdurman, 10 November 1954, *Jackson 3229* (K).

TANZANIA (selected specimens): **Arusha region**: Lake Manyara, 15 June 1965, *Greenway & Kanuri 11860* (K); **Dodoma region**: Bahi, 3000 ft, 23 April 1962, *Polhill & Paulo 2132* (K, BR0000017458801); **Kilimanjaro region**: Lake Chala crater rim, 3°19'12"S, 37°40'48"E, 8 January 2004, *A. Hemp 4242* (WU); **Lindi region**: 25 km S of Lindi, 280 m, 3 January 1935, *Schlieben 5926* (BM, P04583538, BR0000017458825); **Mara region**: Serengeti, Kirawira plain, 900 m, 27 April 1965, *Richards 20298* (K; BR0000017458757); **Morogoro region**: Mbangala, 700 m N of Mahenge station, 22 February 1932, *H.J. Schlieben 1821* (P04583522, BR0000017458818); Mahenge, Ubangala, 22 February 1932, *Schlieben 1821* (BM); Tanganyika, Morogoro, 2000 ft, March 1935, *E.M. Bruce 926* (P04583534); Morogoro distr., November 1953, Semsei 1425 (K); Mbulu distr., Tarangire Nat. Park, road to Burungi Lake, 1066 m, 13 November 1970, *M. Richards 25439* (BR0000017458764, M); Mlali valley, Ngerengere, near Madanganya, 550 m, 25 February 1970, *T. Pocs 6135/D* (BR0000017458689); Mbulu distr., Iringa distr., Kidatu, 5 February 1971, *Mhoro 473* (K); **Rukwa region**: Sumbawanga distr., Muse, 29 December 1961, *Robinson 4809* (K); **Ruvuma region**: Songea distr., Mbamba Bay, 460 m, 5 April 1956, *E. Milne-Redhead & P. Taylor 9522* (K, BR0000017458795); **Shinyanga region**: Nyika, July 1894, *R.P. Sacleux 2183* (P04583540); Shynianga, 25 February 1950, *Weloh 26* (K); **Tanga region**: Lushoto distr., 29 April 1953, *Drummond & Hemsley 2274* (K); Muheza distr., 4 May 1987, *Iversen et al. 87197* (K); on the road between Bagamoyo and Msata, 6°25'48"S, 38 54'E, 17 December 2004, *A. Hemp 4222* (WU); **Zanzibar**, May-June 1883, *M.G. Revoil s.n.* (P04583479).

TOGO: **Kara region**: Kpagouda, 10 May 1980, *H. Scholz 870* (K); **Maritime region**: Lome, 1900-1902, *Warnecke 6* (BM, K, P04583527, BR0000017458412); Lillikope, 6°34'N, 1°11'E, 22 July 1977, *F.J. Breteler 7023* (BR0000017458382); Lome to Cacaveli, 14 April 1978, *Hakki et al. s.n.* (B); **Plateaux region**: Klouto [Kloto], 10 September 1973, *Hiepko & Schultze-Motel 150* (K, P04583417); Atakpame, 18 September 1973, *Hiepko & Schultze-Motel 257* (K, P04583416).

UGANDA (selected specimens): **Central region**: Singo co., Wattuba, 13 April 1970, *Katende 103* (K); Mukono, Kyagwe, 20 March 1992, *Rwaburindore 3395* (K); North Buganda, Mukono, Kyagwe, Bule-Buyaga, 0°17'S, 32°42'E, 1200 m, 20 March 1992, *P.K. Rwaburindore 3395* (BR0000017458610); **Eastern region**: Busoga distr., 17 April 1953, *Wood 724* (K); **Kampala**, [no date] *Liebenberg 839* (K); **Western region**: Toro distr., 24 December 1934, *Taylor 2655* (BM); Ruwenzori, *Scott Elliot 6232* (BM).

ZAMBIA: **Central province**: Chitala, 12 February 1959, *Robson 1552* (K); **Eastern province**: near Changwe, between Petauke & Mwape, 650 m, 16 December 1958, *N. Robson 959* (BM, K, BR0000017458870); Luangwa valley, Game Ras, 21 February 1967, *Prince 292* (K); Mopane woodland, North Luangwa National Park, 17 March 1994, *Smith 446* (K); **Southern province**: Victoria Falls, south bank of Zambezi, 12 February 1912, *Rogers 5701* (BM); Kalomo, 16 February 1965, *Fanshawe 9112* (K); Gwembe distr., Changa, 12 February 1973, *Kornash 3258* (K).

ZIMBABWE: **Manicaland province**: Sabi valley, Nyanyadzi, 1800 ft, 3 February 1948, *H. Wild 2494* (BR0000017458917); Nyanga distr., 29 November 1959, *Goodier 686* (K); **Mashonaland West province**: Urungwe, 29 January 1958, *Drummond 5326* (K); **Masvingo province**: Nuanetsi, 8 May 1958, *Drummond 5702* (K); Chibi distr., 29 December 1962, *Moll 358* (E); **Matabeleland South province**: Beitbridge, 22 March 1967, *Mavi 280* (K); Insiza, 8 February 1974, *Mavi 1522* (K); **Midlands province**: Queque [Kwekwe], 1 May 1976, *Dye 473* (K).
